# Detection of Circulating Tumor DNA in Patients with Thyroid Nodules

**DOI:** 10.1155/2021/8909224

**Published:** 2021-08-23

**Authors:** Krupal B. Patel, Nicholas Cormier, James Fowler, Allison Partridge, Julie Theurer, Morgan Black, Nicole Pinto, John Yoo, Kevin Fung, Danielle MacNeil, William Stecho, Christopher Howlett, Muriel Brackstone, John W. Barrett, Anthony Nichols

**Affiliations:** ^1^Department of Otolaryngology-Head and Neck Surgery, Western University, London, Ontario, Canada; ^2^Department of Pathology, Western University, London, Ontario, Canada; ^3^Department of Surgery, Western University, London, Ontario, Canada

## Abstract

**Objective:**

Detection of circulating tumor DNA (ctDNA) in cancer patients can potentially serve as a noninvasive, sensitive test of disease status. The purpose of this study was to determine the ability to detect *BRAF (V600E)* mutations in the plasma of patients with thyroid nodules, with the goal of distinguishing between benign and malignant nodules.

**Methods:**

Consecutive patients with thyroid nodules who consented for surgery were recruited. Plasma samples were obtained preoperatively and one month postoperatively. Quantitative PCR was used to determine the levels of the *BRAF (V600E)* mutation preoperatively and postoperatively.

**Results:**

A total of 109 patients were recruited. On final pathology, 38 (32.8%) patients had benign thyroid nodules, 45 (38.8%) had classical papillary thyroid cancer (PTC), 23 (19.8%) had nonclassical PTC, and 3 (2.6%) had follicular thyroid cancer. 15/109 patients had detectable *BRAF (V600E)* ctDNA in their preoperative samples—all of them having classical PTC. Higher T-stage and extrathyroidal extension in PTC were associated with positive *BRAF (V600E)* ctDNA (*p* < 0.05). Eighty-eight pairs of preoperative and postoperative plasma samples were collected and analyzed. Of these eighty-eight paired samples, a total of 13/88 (14.8%) patients had detectable *BRAF (V600E)* ctDNA in their preoperative samples—all of them having classical PTC. 12 of these 13 patients had no detectable *BRAF (V600E)* postoperatively, while one remaining patient had a significant decline in his levels (*p* < 0.05).

**Conclusion:**

*BRAF (V600E)* circulating thyroid tumor DNA can be detected in plasma and is correlated with a final diagnosis of the classical variant of PTC. Given that a postoperative drop in *BRAF (V600E)* ctDNA levels was observed in all cases suggests its utility as a tumor marker.

## 1. Introduction

Thyroid nodules are common, occurring in 5% of women and 1% of men by palpation and 19–68% on high-resolution ultrasound [1–4]. The majority of nodules are benign, while 7–15% harbor malignancy depending on risk factors [5, 6]. The guidelines for the workup of these nodules by the American Thyroid Association (ATA) suggest a dedicated thyroid ultrasound first followed by a subsequent fine-needle aspiration (FNA) for any nodules that meet appropriate size and imaging characteristics [7]. However, a significant number of patients have insufficient or indeterminate biopsies and undergo diagnostic surgery for a definitive diagnosis. While hemithyroid and total thyroidectomy are routine and generally safe surgical procedures, there are risks including hypothyroidism, transient or permanent hypocalcemia, hematoma, and injury to both superior and recurrent laryngeal nerves.

In order to address these diagnostic shortcomings, two proprietary tests have been developed targeting the intermediate and indeterminate Bethesda categories through molecular testing of additional FNA samples—Afirma and ThyroSeq v2 [8, 9]. However, these tests have significant false positive and false negative rates, particularly when other groups have attempted to externally validate the findings of the initial studies [10–18]. In addition, both of these tests require additional FNA samples, which can cause additional patient pain and anxiety. Ideally, a noninvasive test could provide improved patient comfort while providing similar information to guide patient care.

An additional shortcoming of thyroid diagnostics is thyroid cancer surveillance. Current guidelines for surveillance include serial stimulated and unstimulated thyroglobulin levels, neck ultrasound, and radioactive I^131^ scans in selected cases that receive adjuvant radioactive iodine [19]. However, 23–29% of patients with well-differentiated thyroid cancers express thyroglobulin antibodies thus making surveillance of thyroid cancer recurrence difficult [20–23]. Moreover, 12% of cases are thyroglobulin negative preoperatively with the entire gland *in situ*, highlighting the imperfections of thyroglobulin for monitoring disease burden [24]. A highly accurate blood test that can detect disease relapse would greatly improve care.

With recent advances in molecular technology, there has been great interest in using circulating tumor DNA (ctDNA) in the detection and surveillance of cancer. ctDNA can be released into the bloodstream by apoptotic and necrotic cancer cells, actively secreted by tumor cells or after tumor cells are processed by macrophages [25–27]. Thus, the interrogation of ctDNA plasma or serum can be used as a “liquid biopsy,” circumventing the need for a tissue biopsy, facilitating surveillance of cancer, and can potentially be utilized in various cancer types for detection or surveillance. In order to detect ctDNA, it is necessary to screen for variants present in the primary tumor. In the setting of surveillance, the primary tumor can be characterized, and a particular variant(s) can be selected for analysis. For screening, knowledge of the molecular landscape of the primary tumor type is necessary to design an appropriate assay. Papillary thyroid cancer (PTC) accounts for 70% of thyroid cancers, and 60% of PTCs carry canonical *BRAF (V600E)* mutations [28]. As a consequence, a large number of potential thyroid cancers can potentially be detected by screening for a single mutation. In this pilot study, we aim to prospectively screen for the *BRAF (V600E)* mutations in the plasma of patients undergoing surgery for thyroid nodules to assess the sensitivity and accuracy of ctDNA for the detection of thyroid cancer and to evaluate its value as a tool for thyroid cancer surveillance.

## 2. Methods

### 2.1. Patient Recruitment

Sequential patients referred to the Otolaryngology-Head and Neck Surgery Clinic for thyroid nodules at London Health Sciences Centre (LHSC) from April 2014 to March 2015 were approached for participation in the study. Approval was obtained through the Lawson Health Research Institute research ethics board (REB 103985). Inclusion criteria included patients over the age of 18 and those scheduled to undergo partial or total thyroidectomy for their thyroid nodules. Exclusion criteria included a previous cancer known to be positive for the *BRAF (V600E)* mutation (such as melanoma, lung cancer, and colon cancer).

### 2.2. Specimen Collection and Nucleic Acid Isolation

Patient's blood was collected in 5 mL EDTA-coated blood collection tubes by the LHSC lab, and blood was separated within the hour into plasma and red blood cells following centrifugation at 1000 ×g for 10 minutes at room temperature. A total of 1 mL of plasma was aliquoted into cryovials and frozen at −80°C. Aliquoted plasma samples were thawed and equilibrated to room temperature. The QIAamp circulating nucleic acid kit (Qiagen, cat no. 55114) was used for the isolation of circulating nucleic acids as per manufacturer's instructions.

Formalin-fixed paraffin-embedded (FFPE) samples from the index thyroid nodule of 11 patients who had benign nodular hyperplasia on their final pathology and 20 patients who had classical PTC on their final pathology were retrieved. Cores were obtained, and nucleic acids were extracted using the QIAamp DNA FFPE tissue kit (Qiagen, cat no. 56404). Concentration of the nucleic acids was measured using a NanoDrop 2000 instrument (Thermo Scientific).

### 2.3. Quantitative Polymerase Chain Reaction (qPCR) for *BRAF (V600E)*

Qiagen QuantiTect Multiplex PCR Kit (cat no. 204543) was used for qPCR. A 20 *μ*l reaction with 10 *μ*l of 2x QuantiTect Multiplex PCR Master Mix, 4 *μ*M (0.04 *μ*l of 100 *μ*M) of each primer, and probe was used. RNAse-free water was used to bring the final volume of each reaction to 20 *μ*l. 100 ng (0.2 *μ*l) of the DNA template was used in each 20 *μ*l reaction. Each reaction included the primer-probe set for BRAF nonmutated exon (exon 6) of the gene and *BRAF (V600E)*-mutated gene. qPCR cycling conditions were as follows: initial activation step of 15 min at 95°C, denaturation for 1 min at 94°C, and annealing/extension for 90 sec at 62°C for 40 cycles. Each sample was replicated at least twice and done in duplicate each time to account for intra-assay and interassay variations. Extracted DNA from selected samples which were positive and negative for the *BRAF (V600E)* mutation was sent for Sanger sequencing to confirm the qPCR findings. Primer/probe sequences were custom designed, and experimental conditions were optimized (see Supplemental Table 1 for the primer/probe sequences).

Positive and negative controls were used to determine the cycle threshold to account for interassay variations. A dilution curve using standards was then used to calculate the relative copy number of *BRAF (V600E)* using the formula *BRAF (V600E)*/*BRAF* wild type.

### 2.4. Statistical Analysis

Statistical analysis was done using GraphPad Prism 7 (GraphPad Software Inc., CA). Student's *t*-test was used to compare preoperative and postoperative *BRAF (V600E)* ctDNA levels. Fischer's exact test was used to determine the association between detectable *BRAF (V600E)* ctDNA and clinicopathologic characteristics.

## 3. Results

### 3.1. Patient Characteristics

**Figure 1:**
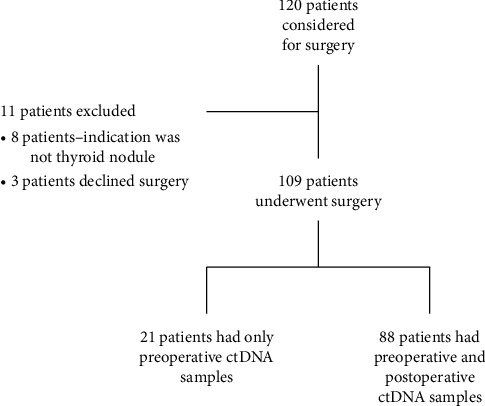
Patient recruitment—inclusion and exclusion.

A total of 109 consecutive patients who consented for surgery for thyroid nodules were prospectively recruited. 36 (33%) were males and 73 (67%) were females (see Table 1 and Figure 1 for patient characteristics). Of these 109 patients, based on preoperative fine-needle aspiration biopsies, 2 of the nodules (1.8%) were Bethesda I, 24 (22%) were Bethesda II, 23 (21.1%) were Bethesda III, 21 (19.3%) were Bethesda IV, 9 (8.3%) were Bethesda V, and 30 (27.5%) were Bethesda VI. Of these 109 patients, based on the final pathology report, 38 (32.8%) of the nodules were benign, 45 (38.8%) were classic PTC, 23 (19.8%) were nonclassic histologic variants of PTC, and 3 (2.6%) were follicular thyroid cancer (FTC). Eighty-eight of 109 patients (80.7%) had both preoperative and postoperative ctDNA samples collected. Of these eighty-eight paired samples, final pathology indicated that 28 (31.8%) patients had benign thyroid nodules, 38 (43.2%) had classical papillary thyroid cancer (PTC), 19 (21.6%) had nonclassical PTC, and 3 (3.4%) had follicular thyroid cancer.

### 3.2. *BRAF (V600E)* ctDNA Was Only Detected in Patients with Classical PTC

A total of 15/109 (13.8%) patients had detectable *BRAF (V600E)* in the preoperative plasma samples (Table 1), all of which had classical PTC as the final pathologic diagnosis. Of the 15 patients with detectable *BRAF (V600E)* in the preoperative plasma samples, preoperative fine-needle aspiration biopsies indicated the following distribution: 1 (6.7%) patient had Bethesda II, 4 (26.7%) patients had Bethesda III, 1 (6.7%) patient had Bethesda IV, 3 (20%) patients had Bethesda V, and 6 (40%) patients had Bethesda VI. No patients with nodular hyperplasia, nonclassical PTC, or FTC had *BRAF (V600E)* ctDNA.

### 3.3. Correlation of *BRAF (V600E)* ctDNA with Classic PTC Staging

12 out of 15 (80%) patients who had detectable *BRAF (V600E)* ctDNA with the final diagnosis of PTC had advanced T-stage (T3-4) compared with patients with undetectable levels (*p* < 0.05) (Table 2). Additionally, 10/15 (6.7%) patients who had detectable *BRAF (V600E)* ctDNA had extrathyroidal extension (ETE), and this was significantly higher in proportion than patients with undetectable ctDNA levels (*p* < 0.05). Nodal staging was not correlated with *BRAF (V600E)* ctDNA levels.

### 3.4. *BRAF (V600E)* Declined after Surgery in All Patients with Detectable Baseline Levels

**Figure 2:**
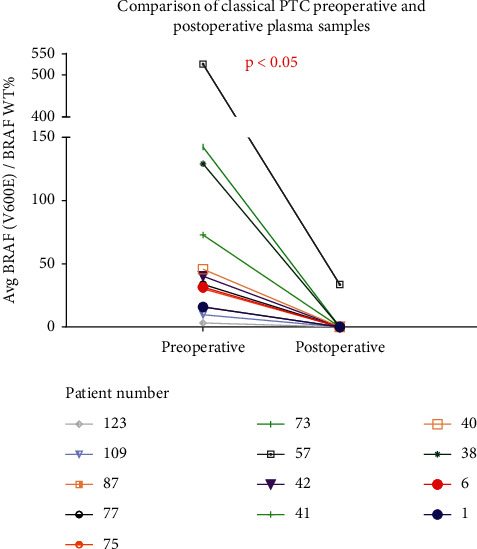
*BRAF (V600E)* ctDNA detection preoperatively and postoperatively in patients with classical PTC pathology. ^*∗*^13/38 (31.6%) patients had detectable levels preoperatively, and all declined postoperatively. ^*∗*^Those patients with detectable levels of *BRAF (V600E)* ctDNA are shown.

Eighty-eight patients were followed postoperatively with an additional blood draw at one month follow-up. Thirteen of 88 patients (14.8%) had detectable *BRAF (V600E)* in preoperative plasma samples—all of them classical PTC patients, and all of them decreased postoperatively (*p* < 0.05), with twelve patients having nondetectable *BRAF (V600E)* postoperatively (Figure 2). Only one patient who was found to have unresectable disease and had gross disease left behind had a postoperative detectable ctDNA level (Figure 2).

### 3.5. Comparison of Preoperative Plasma and FFPE Samples

To assess the concordance between preoperative *BRAF (V600E)* ctDNA and *BRAF (V600E)* mutational status of the index thyroid nodule, a subset of 31 FFPE samples was obtained, and mutational status for *BRAF (V600E)* was determined. Eleven samples had a final diagnosis of nodular hyperplasia, and 20 samples had a final diagnosis of classical PTC. Of the 11 nodular hyperplasia samples, none had detectable *BRAF (V600E)* in their ctDNA; however, 2/11 (18.2%) did have *BRAF (V600E)* mutational status in their FFPE samples (Table 3). For the classical PTC samples, discordances in mutational status were surprisingly noted in 12/20 (60%) cases (Table 3).

## 4. Discussion

In our pilot study, we successfully detected *BRAF (V600E)* ctDNA in 15 (13.8%) out of the 109 patients with thyroid nodular disease selected for surgery, all of which had classical PTC in the index nodule. Comparing the pre- and postoperative plasma samples, all patients who had detectable *BRAF (V600E)* ctDNA at baseline experienced a significant decline in levels at one month postoperatively. Twelve of the 13 cases declined to nondetectable levels, while one of the patients (patient 57) had a significant decline, but it was still detectable. This particular patient had undergone a total thyroidectomy, right central neck dissection, and right neck dissection for this disease. Intraoperatively, the recurrent laryngeal nerve was encased in the tumor and had positive margins on the final pathology. Postoperative SPECT/CT after radioactive iodine treatment showed two focal areas of uptake within the thyroid bed suggesting residual disease. The fact that ctDNA levels remained positive in this case with clear residual disease and negative in all others suggests that ctDNA may play a useful role in postoperative surveillance. A larger sample size and longer follow-up are necessary to draw more definitive conclusions.

Circulating tumor DNA is a noninvasive test that has a demonstrated ability to identify subclinical disease and recurrence prior to clinical detection across multiple cancers [29–33]. In thyroid cancer, there are preliminary data suggesting that it may play a similar role [34–39]. *BRAF (V600E)* was chosen for this pilot study as it is the most common genetic alteration noted in PTCs, which is also the most common type of well-differentiated thyroid cancer [28]. There have been varying reports of the detection of *BRAF (V600E)* ctDNA in plasma of patients with thyroid cancer. Pupilli et al. studied 103 patients with thyroid nodules with preoperative and postoperative blood testing. Seventeen patients had positive ctDNA levels preoperatively, twelve of which (71%) became undetectable *BRAF (V600E)* ctDNA postoperatively. The remaining 5 patients had detectable levels postoperatively, but they were significantly lower compared to the baseline levels [34]. The higher detection rate in their cohort is likely due to patient selection bias as 12/17 had RAI postoperatively. Cradic et al. reported *BRAF (V600E)* ctDNA in 20 (11.6%) of the 173 thyroid cancer patients correlating with the presence of active disease at the time of the blood draw [36]. Chuang et al. found that 3/14 (36%) matched tumor and serum patients with PTC had *BRAF (V600E)* ctDNA preoperatively. [35]. Kim et al. found *BRAF (V600E)* ctDNA in only 6.1% of the patients (3/49) with all three patients having lateral lymph node or lung metastasis [37]. A recent study conducted by Allin et al. and Lubitz et al. indicated the value of *BRAF (V600E)* ctDNA in the surveillance of advanced thyroid cancers and earlier detection of disease progression [38, 40].

In our study, none of the patients with nodular hyperplasia on final pathology had *BRAF (V600E)* ctDNA. Previous reports have suggested that up to 13.3% (2 out of 15) of thyroid nodules determined to be benign on final pathology can harbor *BRAF (V600E)* mutations [41]. It has been speculated that these *BRAF (V600E)*-positive “benign” thyroid nodules may in fact be premalignant [42]. Interestingly, in our study, 2 out of 11 patients (18.2%) with benign nodular hyperplasia did have the *BRAF (V600E)* mutation in the index thyroid nodule FFPE samples. Similarly, we observed poor concordance between the plasma and FFPE BRAF mutational status in the classical PTC samples with only 40% concordance (Table 3). This discrepancy, while surprising, has been reported in other studies with concordance rates ranging from 11% to 60% [34–36, 39]. One possible explanation for this is that thyroid cancer is often a multifocal disease with tumor heterogeneity [43]. Although *BRAF (V600E)* ctDNA is detected in plasma, the corresponding index thyroid nodule FFPE sample is negative for the mutation as the sample may have been obtained from a nodule or portion of a cancer focus that did not harbor the mutation. It has been demonstrated that *BRAF (V600E)* can be acquired as a secondary change during tumor progression or it might be limited to subclonal populations or separate foci in a multifocal tumor [42, 44]. Additionally, it is important to remember that *BRAF (V600E)* ctDNA can be positive in plasma in other malignancies as well such as melanoma, lung cancer, and colorectal cancer [29, 45]. Another important consideration in liquid biopsies is clonal hematopoiesis (CH) [46]. CH is a process by which there is accumulation of somatic mutations in the hematopoietic stem cells leading to clonal expansion of mutations in blood cells [47–50]. These somatic mutations originating from the blood cells can lead to false positive interpretation—i.e., somatic mutations detected in blood are misattributed to originating from the primary tumor when, in fact, they are originating from blood cells [51, 52]. CH can also account for decreased concordance between the primary tumor and ctDNA as noted by Razavi et al., where only 24.4% of the somatic mutations identified in plasma DNA also existed in matched tumors [49]. The effect of CH can be studied by using peripheral blood cells as controls to assess the origin of somatic mutations.

Conversely, the index nodule may harbor the BRAF mutation but does not happen to shed significant *BRAF (V600E)* DNA due to biological factors including tumor size, invasion, and nodal metastases resulting in negative ctDNA levels. This has been noted in other solid tumors where higher disease burden solid tumors are more likely to shed tumor-derived DNA into their bloodstream [53–56]. Indeed, in our study, we demonstrated a correlation of ctDNA levels with advanced T-stage and extrathyroidal extension supporting this hypothesis.

Although this is the first report demonstrating that *BRAF (V600E)* ctDNA was correlated with higher T-stage in patients with classical PTC, the *BRAF (V600E)* mutation in PTC has been shown to correlate with poorer prognosis [57]. Tufano et al. included 14 studies in their meta-analysis to assess the prognosis of PTC in the presence of *BRAF (V600E)* [58]. Risk ratios in *BRAF (V600E)*-positive patients were 1.93 for PTC recurrence, 1.32 for lymph node metastasis, 1.71 for ETE, 0.95 for distant metastasis, and 1.70 for advanced stage AJCC III/IV. These facts highlight another potential role for BRAF ctDNA as a prognostic tool; however, larger prospective cohorts will be necessary to draw definitive conclusions.

A significant limitation of our study is the inclusion of a single point mutation. Although *BRAF (V600E)* mutations are the most frequent alteration in PTC, thyroid cancers can also be driven by point mutations in RET, RAS, EIF1AX, TP53, kinase gene fusions, and arm-level chromosome changes [28]. Moving forward, a next-generation sequencing panel, similar to the approach used in the ThyroSeq panel, could be utilized on plasma instead of our RT-PCR platform to capture these additional changes [9]. An attempt at this has been reported by Lupo et al.; however, their results indicated that their panel was neither sensitive nor specific enough over the panel testing of FNA material [59]. This approach could potentially make the assay much more accurate for discriminating benign from malignant disease and serve as an effective tumor surveillance marker for a larger proportion of thyroid cancers. This is a rich avenue for further research.

## 5. Conclusion

In summary, our study shows that *BRAF (V600E)* ctDNA can potentially be used as a marker of aggressive disease and as a surveillance marker in a subset of thyroid cancers. Further work is needed to delineate its utility to differentiate between malignant and benign thyroid nodules.

The current study only included *BRAF (V600E)* mutations; however, a host of additional driver mutations including point mutations, copy number variations, and translocations have been identified in thyroid cancer [28]. The addition of more genes, along with the use of more sensitive ctDNA detection techniques, will be beneficial for future studies.

## Figures and Tables

**Table 1:** Preoperative patient characteristics.

Preoperative samples (*n* = 109)			Preoperative ctDNA positivity (*n* = 15)
	Males	36 (33%)	
	Females	73 (67%)	

Pathology	Benign	38 (32.8%)	0 (0%)
	Classical papillary thyroid cancer (PTC)	45 (38.8%)	15 (100%)
	PTC, nonclassical	23 (19.8%)	0 (0%)
	Follicular thyroid cancer	3 (2.6%)	0 (0%)

**Table 2:** Correlation of *BRAF (V600E)* ctDNA and T-stage, N-stage, and ETE.

		ctDNA negative	ctDNA positive	*p* value^a^
T-stage^b^	Low T-stage	16	3	*p* < 0.05
	High T-stage	14	12	

N-stage^c^	0	20	6	*p* > 0.05
	1	10	9	

ETE^d^	Absent	21	5	*p* < 0.05
	Present	9	10	

^a^Fischer's exact test was used to assess for statistical significance. ^b^Low T-stage refers T1 or T2; high T-stage refers T3 or T4. ^c^N-stage: nodal staging. ^d^ETE: extrathyroidal extension.

**Table 3:** Concordance between index nodule FFPE pathology and *BRAF (V600E)* ctDNA.

	FFPE tissue	ctDNA negative	ctDNA positive
Nodular hyperplasia	Negative	9	0
	Positive	2	0

Classical PTC	Negative	3	2
	Positive	10	5

## Data Availability

The datasets used and/or analyzed during the current study are available from the corresponding author upon reasonable request.

## Abbreviations

cfDNA: Cell-free DNA
ctDNA: Circulating tumor DNA
ETE: Extrathyroidal extension
FFPE: Formalin-fixed paraffin-embedded
FNAB: Fine-needle aspiration biopsy
FTC: Follicular thyroid cancer
N-stage: Nodal stage
PTC: Papillary thyroid cancer
qPCR: Quantitative polymerase chain reaction
T-stage: Tumor stage
US-FNAB: Ultrasound-guided fine-needle aspiration biopsy.

## Supplementary Materials

Supplementary Materials Supplemental Table 1: primer/probe sequences for the *BRAF* nonmutated exon and *BRAF (V600E)*.
