# Supplementary material for: Detection of Circulating Tumor DNA in Patients with Thyroid Nodules
Source: Int J Endocrinol. 2021 Aug 23;2021:8909224. doi: 10.1155/2021/8909224 (PMC8407979; doi:10.1155/2021/8909224)
Supplement: Supplementary Materials — Supplemental Table 1: primer/probe sequences for the BRAF nonmutated exon and BRAF (V600E). [file 8909224.f1.docx]

**Supplemental Table 1: Primer/probe sequences for *BRAF* non-mutated exon and *BRAF(V600E)***

| ***BRAF non-mutated exon*** | Forward Primer | 5’-TAGGTGATTTTGGTCTAGCTACCGA |
| --- | --- | --- |
|  | Reverse Primer | 5’-GGATCCAGACAACTGTTCAAACTG |
|  | Probe | 5’-[JOE]GAATCTCGATGGAGTGGGTC |
| ***BRAF(V600E)*** | Forward Primer | 5’-GATGCACTCCAACAAAGAGAACAA |
|  | Reverse Primer | 5’-GGTATCCATTGATGCAGAGCTAGA |
|  | Probe | 5’-[ROX]TCTCTGGGGAACGGAACTGA |
